# Synthesis and Characterization of Naringin Phenolate Salts for Cultivated Meat

**DOI:** 10.1021/acs.jafc.6c09167

**Published:** 2026-09-24

**Authors:** Matilda Dirawy, Muhammad Abdel-Haq, Sarah E. Hasenson, Sara Mahajna, Mohamed Mahameed, M. N. V. Ravi Kumar, Meenakshi Arora, Abraham J. Domb

**Affiliations:** † Institute for Drug Research, School of Pharmacy, Faculty of Medicine, 26742 The Hebrew University of Jerusalem, Jerusalem 91120, Israel; ‡ SuperMeat Co., The Essence of Meat Ltd, Pinhas Sapir 3, Nes Ziona 7403626, Israel; § Department of Translational Science and Medicine, College of Community Health Sciences and the Center for Convergent Bioscience and Medicine (CCBM), 8063 The University of Alabama, Tuscaloosa, Alabama 35401, United States

**Keywords:** Cultivated meat, Polyphenols, Naringin salts, Cell proliferation

## Abstract

The high cost of growth factors has limited the commercial-scale production of cultivated meat products. In this study, we report on the synthesis and characterization of a series of naringin phenolate salts. Phenolate salts with sodium (Na^+^), potassium (K^+^), calcium (Ca^2+^), and tetrabutylammonium (TBA^+^) counterions were prepared to introduce water solubility to the natural flavonoid naringin. The synthesized salts were comprehensively characterized to confirm their chemical structures. Solvated cations were examined for their effect on the salts’ physicochemical and biological properties. Solubility studies show that alkali metal salts are highly soluble in water, while the Ca^2+^ and TBA^+^ salts are less soluble. Acid regeneration studies confirm the reversibility of the salt formation. Preliminary studies indicate that selected naringin salts may support cell proliferation in cultivated meat systems. Overall, this study presents the first comprehensive report on naringin phenolate salts and their potential use in cultivated meat production.

## Graphical abstract

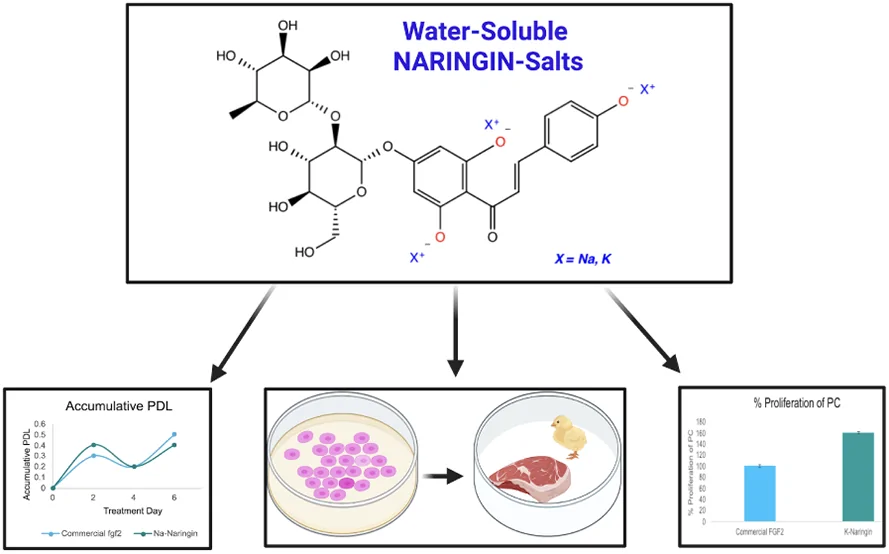

## 1 Introduction

Naringin is a naturally occurring polyphenolic secondary metabolite predominantly found in citrus fruits, particularly grapefruits and oranges.^1^ Chemically, it is a glycosylated derivative of the flavonoid naringenin, formed by the attachment of the disaccharide neohesperidose (rhamnose–glucose) to the C-7 hydroxyl group of naringenin^2–4^ (Figure 1). Due to the unique position of this glycosidic linkage, naringin has attracted considerable scientific interest in recent years, leading to reports that naringin exhibits anti-inflammatory, antioxidant, hypolipidemic, and anticancer effects, among others.^5–7^ These biological effects, combined with its natural abundance and favorable safety profile, have made naringin a promising candidate for the development of novel therapeutic and nutraceutical formulations. Cultivated meat is part of an emerging technology for producing meat in a more sustainable way. One of the aspects of cultivated meat production is the scale-up of cell growth by using growth-promoting factors (GPFs).^8^ Despite biological advantages, the use of growth factors such as Fibroblast Growth Factor 2 (FGF2) remains impractical for large-scale cultivated meat applications due to its high cost, dosage requirement, and short shelf life.^9–12^ Studies show that polyphenols may interact with pathways mediated by Fibroblast Growth Factor Receptors (FGFRs), partially recapitulating the effect of FGF2 and representing a more stable, cost-effective means of supporting cell growth in a serum-free culture.^13,14^ In our recent report, we show that polyphenol salts, including naringenin and quercetin derivatives, do mimic the activity of growth factors.^14^ Specifically, potassium naringenin (K-Ng) and sodium quercetin (Na-Q) were shown to promote aggregate growth in serum-free culture in the absence of FGF2.^14^ Inspired by these findings and considering that naringin is a glycosylated derivative of naringenin, we hypothesize that naringin and its salt derivatives may promote cell proliferation for cultivated meat.^15^

**Figure 1:**
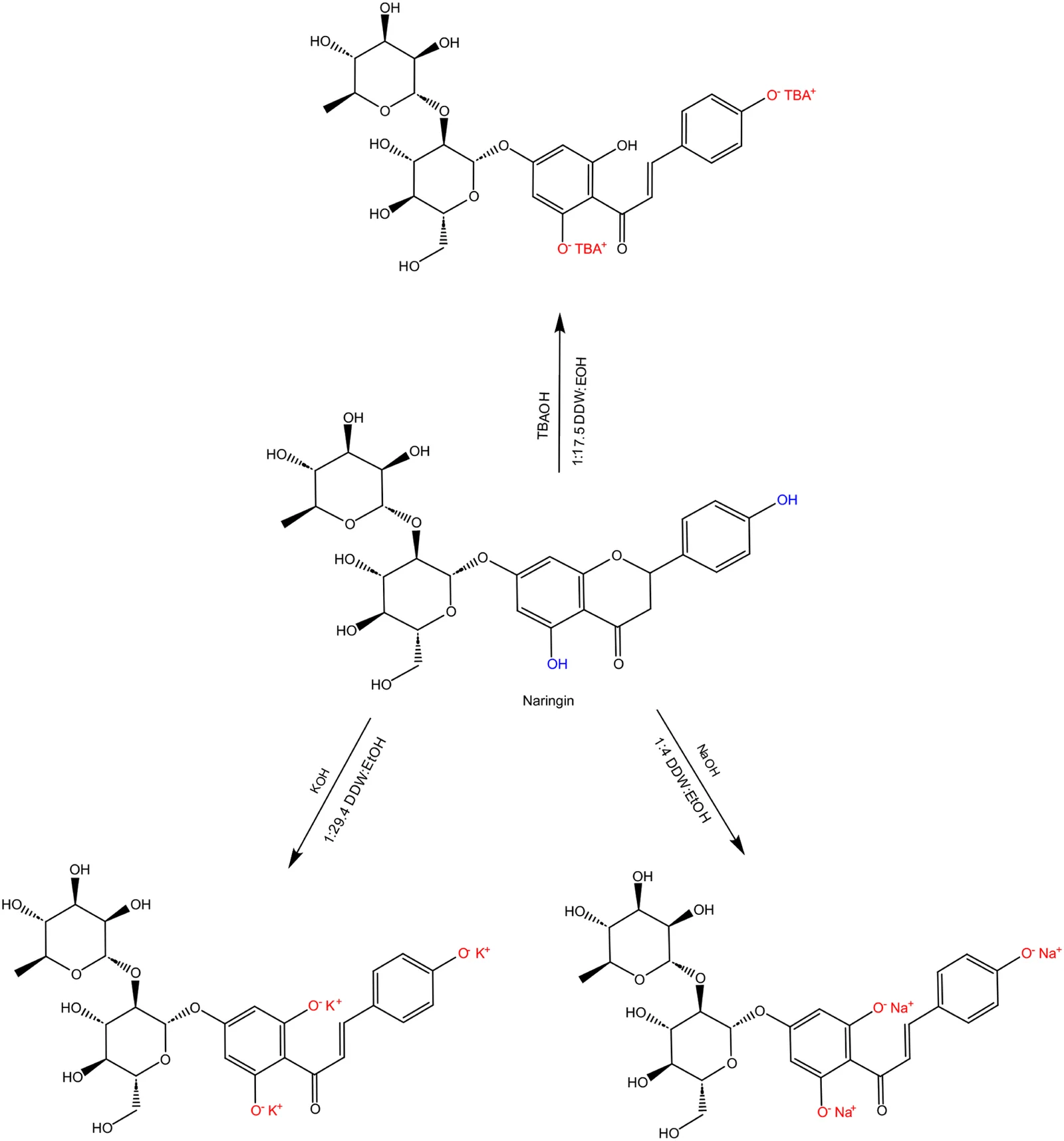
Preparation of naringin salts via reaction with corresponding aqueous and alcoholic bases.

Despite the numerous biological activities attributed to naringin, poor solubility of around 0.5 mg/mL in water, together with its low bioavailability, limit its further utilization in clinical applications.^16,17^ Various strategies, including prodrug design, chemical modification, particle size reduction, and advanced drug delivery systems, have been explored in the literature to improve the solubility and bioavailability of drugs.^18^ While these approaches have shown promise, their widespread application is often limited by challenges related to scalability, high manufacturing costs, formulation complexity, and potential toxicity concerns. In recent years, our laboratory has developed a facile and economical approach for enhancing the aqueous solubility of polyphenolic compounds through their conversion into phenolate salts.^19–22^ The current study represents a significant iteration and advancement of that established methodology. Phenolic groups are weakly acidic in nature. Upon deprotonation, their conjugate bases (phenolate ions) are stabilized through resonance delocalization of the negative charge.^23^ This structural feature enhances their polarity and aqueous solubility, which may, in turn, improve their bioavailability and biological activity. Since naringin is a naturally occurring dietary flavonoid widely consumed via citrus fruits, its derived alkali metal salts present a highly viable pathway toward achieving Generally Recognized as Safe (GRAS) status.^24,25^ This regulatory feasibility has a crucial advantage for the seamless integration of these cost-effective media components into commercial-scale food production systems.

In the present study, naringin phenolate salts (Na^+^, K^+^ , Ca^2+^ and TBA^+^) were synthesized by the reactions of naringin with their corresponding hydroxides (NaOH, KOH, Ca­(OH)_2_, or TBAOH) in either aqueous or alcoholic medium. While phenolate salt formation has been reported for simple phenolic compounds, studies involving structurally complex polyphenols, such as naringin, remain limited. Because of its multiple hydroxyl groups and glycosidic moieties, naringin is an interesting candidate for salt formation and functional modification.

This study synthesizes and comprehensively characterizes naringin salts and evaluates their physicochemical properties, with particular emphasis on aqueous solubility and stability. Structural and thermal analyses were performed on the synthesized salts using proton nuclear magnetic resonance (^1^H NMR), Fourier-transform infrared spectroscopy (FT-IR), elemental analysis, and thermogravimetric analysis (TGA). Acid regeneration studies were also performed to confirm the reversibility of salt formation. In addition, the solubilities of the salts were assessed in various aqueous media.

Following comprehensive chemical characterization, the salts were evaluated for cytocompatibility using a 3-(4,5-dimethylthiazol-2-yl)-2,5-diphenyltetrazolium bromide (MTT) assay. Finally, the synthesized salts were evaluated for their ability to stimulate the proliferation of embryonic chicken stem cells through cell counting, cell viability analysis, and population doubling level (PDL) calculations. These preliminary experiments serve as a proof of concept to assess the potential of the naringin salts to support cell proliferation.

## 2 Methods and Materials

### 2.1 Materials

Naringin (97% purity, CAS no. 10236-47-2) was purchased from Thermo Scientific (Waltham, MA, USA) and used as received. Sodium hydroxide pearls (AR grade, CAS no. 1310-73-2) and potassium hydroxide flakes (AR grade, CAS no. 1310-58-3) were purchased from Bio-Lab Ltd. (Jerusalem, Israel). Calcium hydroxide (purity >96%, CAS no. 1305-62-0) and tetrabutylammonium hydroxide solution (40% in water, CAS no. 2052-49-5) were obtained from Sigma-Aldrich (St. Louis, MO, USA). All of the alkaline reagents were used without further purification. Human FGF2 was purchased from PeproTech (cat number AF-100-18B).

### 2.2 Naringin Salt Formation

Na, K, Ca, and TBA polyphenolate salts were synthesized by treating free naringin with their respective bases (Figure 1). Formation of the salts was monitored via thin-layer chromatography (TLC) and UV–Vis spectroscopy, observing the emergence of a colored precipitate. The consumption of unreacted starting material was quantified at distinct intervals using UV–Vis spectroscopy against a standard calibration curve of free naringin.

#### 2.2.1 Synthesis of Na-Naringin

Sodium hydroxide solution (20% NaOH, 3.4 mL, 0.684 g, 17.11 mmol, 4 equiv) was added dropwise to naringin (2.560 g, 4.277 mmol, 1 equiv) and dissolved in 100 mL of ethanol. The reaction mixture was stirred at room temperature for 24 h to form a precipitate that was isolated by centrifugation and decantation. The collected orange solid was washed with ethanol and acetone, then dried. Yield: 94.5%. FTIR (νmax/cm^–1^): 2920 (ν C–H aliphatic) 1597–1539–1504–1429 (ν C=C) 1259–1169 (ν C–O), 834 (C–H bending). ^1^H NMR (500 MHz, DMSO-d6) δ 8.90 (d, J = 15.8 Hz, 1H), 7.53–7.42 (m, 3H), 6.93 (s, 2H), 5.47 (s, 1H), 5.23 (s, 2H), 5.09 (d, J = 1.5 Hz, 1H), 5.03 (s, 1H), 4.87 (d, J = 7.1 Hz, 1H), 4.59 (s, 5H), 3.81 (dq, J = 9.5, 6.2 Hz, 1H), 3.72–3.65 (m, 2H), 1.19 (d, J = 6.2 Hz, 3H). Elemental analysis calculated (found) (%) for C_27_H_29_O_14_Na_3_ X 5.5H_2_O: C 43.48 (43.11), H 5.37 (5.33), O 41.88 (40.90).

#### 2.2.2 Synthesis of K-Naringin

Potassium hydroxide dissolved in 5 mL of water (0.174 g, 3.102 mmol, 3eq) was added dropwise to naringin (0.6 g, 1.034 mmol, 1 equiv) and dissolved in 20 mL of ethanol. The reaction mixture was stirred at room temperature for 24 h to form a precipitate that was isolated by centrifugation and decantation. The precipitate (the salts) was washed twice with 10 mL of ethanol and 5 mL of acetone to yield a dry yellow powder (83.5%). FTIR (νmax/cm^–1^): 2920 (ν C–H aliphatic) 1596–1539–1502–1425 (ν C=C) 1255–1171 (ν C–O), 834 (C–H bending). ^1^H NMR (500 MHz, DMSO-d6) δ 8.84 (d, J = 15.7 Hz, 1H), 7.45 (d, J = 16.3 Hz, 3H), 6.82 (s, 2H), 5.40 (s, 1H), 5.16 (s, 1H), 5.09 (d, J = 1.5 Hz, 1H), 4.86 (d, J = 7.0 Hz, 1H), 4.60 (s, 4H), 3.85–3.77 (m, 1H), 3.72–3.64 (m, 2H), 3.52 (dd, J = 11.8, 4.2 Hz, 2H), 1.18 (d, J = 6.2 Hz, 3H). Elemental analysis calculated (found) (%) for C_27_H_29_O_14_K_3_ X 4H_2_O: C 42.23 (42.45), H 4.83 (5.08), O 37.58 (36.52).

#### 2.2.3 Synthesis of TBA-Naringin

Tetrabutylammonium hydroxide solution (40% TBAOH, 2.86 mL, 6.615 mmol, 2eq) was added dropwise to naringin (1.280 g, 2.205 mmol, 1 equiv) and dissolved in 50 mL of ethanol. The reaction mixture was stirred at room temperature for 24 h. After completion, the reaction was evaporated using a rotavapor. Yield: 83.4%. FTIR (νmax/cm^–1^): 2961–2874 (ν C–H aliphatic) 1614 (ν C=O) 1581–1533–1498–1461 (ν C=C) 1222–1152–1075 (ν C–O),1320 (OH Bending) 712–737–840–881–988 (C–H bending). ^1^H NMR (500 MHz, DMSO-d6) δ 8.57 (d, J = 15.5 Hz, 1H), 7.34 (s, 1H), 7.06 (d, J = 8.4 Hz, 2H), 6.07 (d, J = 8.3 Hz, 2H), 5.22 (d, J = 2.5 Hz, 1H), 5.12 (s, 1H), 4.96 (d, J = 2.4 Hz, 1H), 4.76 (d, J = 7.4 Hz, 1H), 3.81 (dt, J = 12.5, 6.3 Hz, 1H), 3.68–3.46 (m, 2H), 3.44–3.29 (m, 3H), 3.28–2.98 (m, 24H), 1.54 (td, J = 11.6, 9.9, 5.9 Hz, 23H), 1.30 (h, J = 7.4 Hz, 23H), 1.16 (d, J = 6.1 Hz, 3H), 1.05 (t, J = 7.4 Hz, 0H), 0.92 (t, J = 7.3 Hz, 34H). Elemental analysis calculated (found) (%) for C_27_H_30_O_14_(C_16_H_36_N)_1.5_

*4H_2_O: C 60.41 (59.84), H 9.08 (9.01), O 28.43 (21.80), N 2.08 (2.22)).

#### 2.2.4 Synthesis of Ca-Naringin

Calcium hydroxide powder Ca­(OH)_2_ (249 mg, 3.3075 mmol) was added to a naringin dispersion in water (1280 mg dissolved in 50 mL, 2.205 mmol, 1 equiv). The reaction mixture was stirred at room temperature for 24 h. After completion, the reaction was dried by lyophilization for 72 h. Afterward, the product was washed with 20 mL of ethanol and acetone to remove starting material residues. The final product was dried in an oven (80 °C) overnight. Yield: 81.4%. FTIR (νmax/cm^–1^): 1597–1540–1505–1429 (ν C=C) 1070–1173 (ν C–O), 835–743–702 (C–H bending). Elemental analysis calculated (found) (%) C_27_H_31_O_14_CaOHCa_2_X2H_2_O: C 43.08 (43.11), H 4.78 (4.82), O 36.22 (36.84).

### 2.3 Analytical Methods

^1^H NMR spectra were obtained at 500 MHz (Bruker Avance III) with samples dissolved in DMSO-*d*_6_ and D_2_O. FTIR was performed on a Nicolet iS10 spectrometer (Thermo Fisher Scientific, Waltham, MA, USA) using a Smart iTR sampling accessory. Elemental analysis of C, H, N, and O was performed using the Thermo Flash 2000 CHN-O Elemental Analyzer (Thermo Fisher Scientific, Waltham, MA, USA). UV–vis analysis was performed by UV–vis Ultrospec 2100 pro. Thermal analysis was performed using an Automated and Robotic TGA/DSC 3^+^ system by METTLER TOLEDO, under a nitrogen flow at a rate of 20 mL/min. Samples were heated in 40 μL aluminum pans from 25̊C to 500̊C at a rate of 10̊C/min.

Melting point measurement: The melting points of naringin and the synthesized compound were determined by placing a small amount of the powdered sample into a capillary tube and measuring the melting temperature using a COLE-PARMER MP-200D-HR Stuart High-Resolution Digital Melting Point Apparatus (230 VAC).

### 2.4 Acid-Back-Generation Experiments

Naringin content in the synthesized salts was assessed through acid-back-generation experiments. Treatment of the salts with 1 N HCl or acetic acid promoted reprotonation and precipitation of the parent naringin, while the metal ion remained in solution. The regenerated naringin was extracted with ethanol and quantified by UV–visible spectroscopy, using calibration curves in ethanol solution (Figure S1, Supporting Information).

### 2.5 MTT Assay

Cell viability was evaluated using the 3-(4,5-dimethylthiazol-2-yl)-2,5-diphenyltetrazolium bromide (MTT) assay (Thermo Fisher Scientific), which measures cellular metabolic activity as an indicator of viable cells. Human hepatocellular carcinoma cells (HepG2) were seeded onto the flat-bottomed, 96-well plates at a density of 1 × 10^4^ cells per well in 100 μL of complete culture medium and allowed to adhere overnight. Cells were subsequently treated with naringin (200 μM) for 24 and 48 h. Following treatment, the culture medium was replaced with 100 μL of fresh medium, and 40 μL of MTT solution (1.25 mg/mL in phosphate-buffered saline. PBS was added to each well. Plates were incubated for 5 h at 37 °C to allow the formation of formazan crystals. After incubation, 115 μL of the medium was carefully removed from each well, and 100 μL of dimethyl sulfoxide (DMSO) was added to dissolve the formazan crystals. The plates were then incubated at 37 °C for an additional 10 min with gentle shaking. Metabolic activity was determined by measuring the absorbance at 540 nm, using a BioTek Synergy H1 microplate reader (Agilent Technologies). Metabolic activity was expressed relative to untreated control cells and calculated according to the manufacturer’s instructions. Statistical analysis for the MTT assay was performed using GraphPad Prism software with data from four biologically independent replicates and using a two-tailed, unpaired Student’s *t* test. The data are presented as mean ± standard deviation (SD). The p values are ****P < 0.0001 and *P < 0.05. Cell culture: Human hepatocellular carcinoma cells (HepG2, ATCC: HB-8065) were cultured in Dulbecco’s modified Eagle’s medium (DMEM, Sigma-Aldrich) supplemented with 10% fetal bovine serum (FBS) (Gibco, United Kingdom), penicillin (100 IU/mL), and streptomycin (Diagnovum, Germany), under a humidified atmosphere of 5% CO_2_ in air at 37 °C. Cell viability was calculated by measuring the absorbance of the cells at 540 nm wavelength. MTT was prepared in a stock solution of 1.25 mg/mL in phosphate buffer saline.

### 2.6 Cell Culture and Proprietary Expansion Medium

SuperMeat (SM) proprietary primary embryonic chicken stem cells (EChSCs) were maintained in an SM proprietary serum-free expansion medium optimized for primary cell proliferation in suspension culture. Cells were cultured at 39 °C in a humidified incubator with 5% CO_2_. For standard expansion, cells were passaged every 48 to 72 h. During passaging and reseeding, the final culture volume was adjusted by partial exchange with fresh SM proprietary expansion medium. Compound Treatment and Source: Recombinant human FGF2 was used for control at a concentration of 0.29 nM. The test compounds, sodium naringin (Na-Naringin) and potassium naringin (K-Naringin) were diluted in water and applied at a final concentration of 0.5 nM.

Experimental setup: short-term and long-term suspension culture: Two different culture methods were used for the experimental evaluations. For short-term batch culture experiments (up to 7 days, corresponding to data in Figure 8), cells were cultured in 6-well plates in batch mode without media exchange.

For long-term 12 days studies (corresponding to data in Figure 9), cells were cultured in 125 mL Erlenmeyer flasks with vented caps. The cells were seeded at a standard target density within the range of 1.00 × 10^6^ to 4.00 × 10^6^ cells/mL in fresh medium containing the appropriate experimental compound. These cultures were maintained using a proprietary media-exchange protocol performed every 48 to 72 h. The cells were harvested and counted at each passage.

### 2.7 Assessment of Cell Viability and Proliferation via Flow Cytometry

At harvest, cell counts were determined using a Celldrop automated cell counter. Cells designated for flow cytometry assays were dissociated into single-cell suspensions using TrypLE Express (1X) enzyme for 2 min at 39 °C, followed by filtration through a 70-μm mesh. Cell viability and proliferation were assessed after the indicated number of days of treatment. Cell viability was measured via flow cytometry using 7-aminoactinomycin D (7-AAD) staining. For this assay, harvested cells were incubated with 7-AAD for 5 min at room temperature in the dark before immediate acquisition. Viability data were normalized to the FGF2-treated control set at 100%. Cell proliferation measurements were obtained by staining with Hoechst 34580 for 30 min in the dark at 39 °C. The proliferating fraction was quantified by determining the combined percentage of cells in the S and M phases of the cell cycle (S–M%) using FlowJo analysis software. Proliferation data were normalized to the FGF2-treated control, which was set to 100%. Long-term growth and PDL analysis: Over the two-week study period, cell growth was monitored by quantifying total cell numbers at each passage. Kinetic growth values between consecutive time points were calculated based on absolute cell counts. Accumulative population doubling levels (PDL) were calculated for each passage using the following formula:

$${\mathrm{PDL}}_{\mathrm{new}}=\log10\left(N_{\mathrm{before}}/N_{\mathrm{previous}}\right)/\log10\left(2\right)$$

where:

N_previous_ = the total cell number at the end of the previous passage.

N_before_ = the total cell number before the new passage.

## 3 Results and Discussion

### 3.1 Synthesis of Naringin Salts

Na and K naringin salts were successfully synthesized by reacting with stoichiometric ratios of 1:4 and 1:3 (NaOH and KOH, respectively) in water/ethanol cosolvent systems (1:29.4 v/v for Na; 1:4 v/v for K) at room temperature for 24 h (Figure 1). TBA-naringin salt was prepared by reacting with a 1:2 stoichiometric ratio of naringin and tetrabutylammonium hydroxide in pure ethanol under identical conditions. The calcium (Ca^2+^) salt of naringin was obtained using a 1:1.5 stoichiometric ratio of naringin to calcium hydroxide in water.

### 3.2 Characterization

#### 3.2.1 Proton Nuclear Magnetic Resonance (^1^H NMR) Spectroscopy

The structural integrity and coordination environment of the naringin salts were evaluated using ^1^H NMR spectroscopy in dimethyl sulfoxide-*d*_6_ (DMSO-*d*_6_) (Figure 2). Additionally, spectra for the alkali metal (Na and K) salts were recorded in deuterium oxide (D_2_O) to confirm stability (Supporting Information, Figure S2).

**Figure 2:**
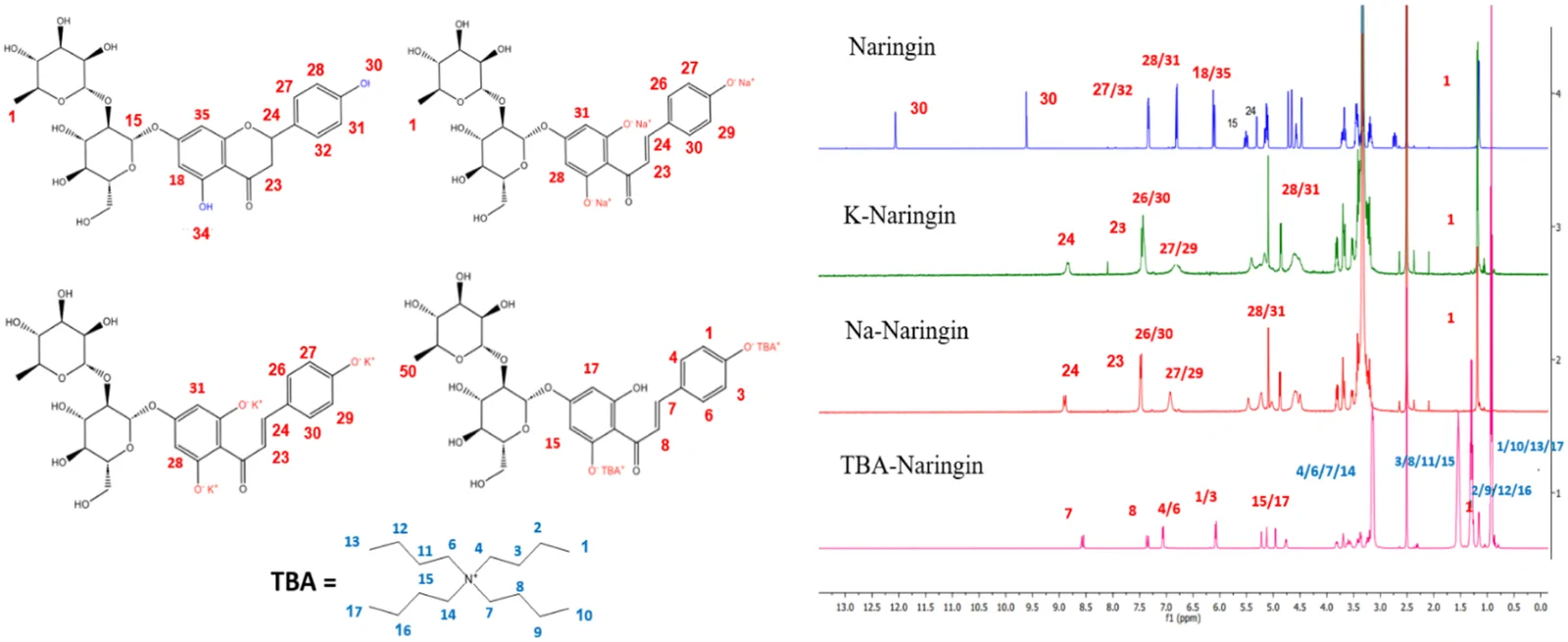
^1^H NMR spectra for naringin and its sodium, potassium, and tetrabutylammonium salts in DMSO-d6 at 500 MHz.

Base-Induced Flavanone—Chalcone Tautomerization: The carbonyl α-carbon protons of naringin have weakly acidic protons, which can be deprotonated under strong basic conditions to generate a resonance-stabilized enolate intermediate that tautomerizes to yield the final product. Treatment of naringin under strong basic conditions (pH > 11) induces a flavonoid-chalcone tautomerization, or the opening of the flavanone ring of naringin, to yield its corresponding chalcone structure. This phenomenon has been well documented in the literature.^26^ The formation of a chalcone structure was confirmed by Heteronuclear Single Quantum Coherence (HSQC) of the product (Figure 3), with both spectra displaying NMR features characteristic of extended conjugated systems. However, for the metal naringin salts that were acidified, the open-chain chalcone structure was found to go intramolecularly back to the flavanone (naringin), showing the reversibility of the interconversion of the two forms at a particular pH. Ring opening introduces a second phenolic hydroxyl group that is also deprotonated by very strong bases, further stabilizing the conjugated system. This is consistent with the spectral observations in the study. The proposed mechanism for the base-induced ring opening and subsequent tautomerization is shown in Figure 4.

**Figure 3:**
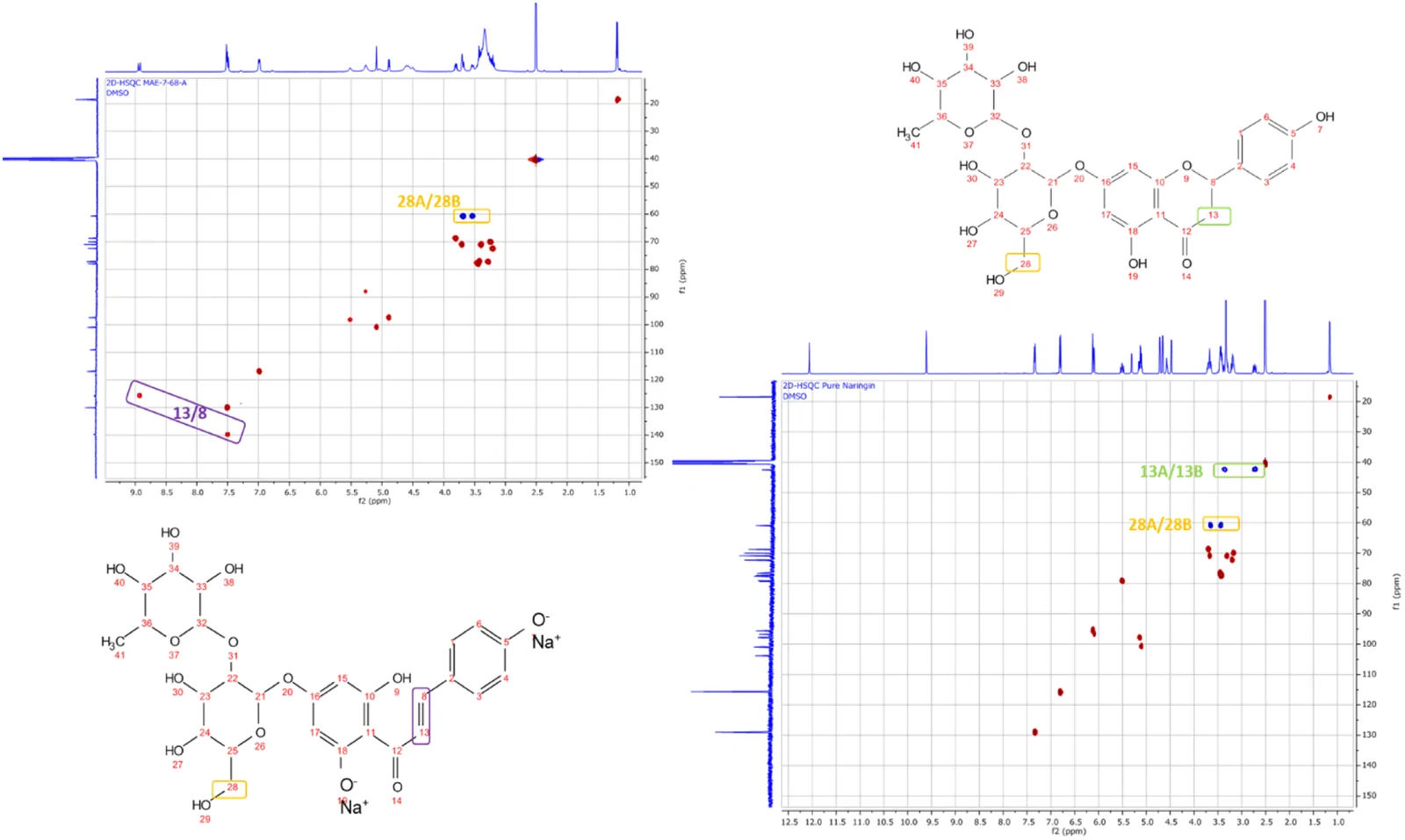
2D-HSQC spectra comparison between naringin and its sodium salt. The spectrum of the sodium salt demonstrates the complete disappearance of the sp^3^ methylene peaks for H-13A/B and the appearance of a downfield peak, confirming ring opening and the formation of a conjugated alpha-beta unsaturated carbonyl system.

**Figure 4:**
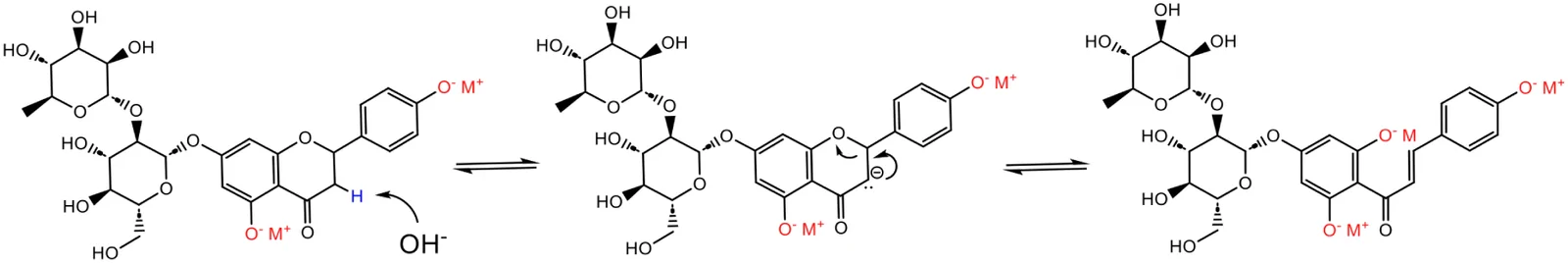
Proposed mechanism for reversible ring opening of soluble naringin salts.

#### 3.2.2 Fourier-Transform Infrared Spectroscopy (FT-IR)

The FT-IR spectra of naringin salts were recorded and compared to those of naringin (Figure S3). In most salts, a reduction in the intensity of the hydroxyl stretching band was observed, which indicates deprotonation. In all of the salts, however, new stretching bands appeared in the hydroxyl region (3200–3600 cm^–1^), suggesting the presence of bound water. This was further confirmed by elemental analysis results. For naringin metal salts, characteristic bands corresponding to the C–H bonds were stretched at 2917–2930 cm^–1^. In the case of TBA naringin, the aliphatic C–H bond stretching was observed at 2961–2874 cm^–1^. In the Na, K, and Ca naringin salts, the disappearance of the C=O stretching band may result from electron delocalization that reduces the double-bond character of the carbonyl group.

#### 3.2.3 Acid Back-Generation and Reversibility Studies

Since phenolate salts are characterized by labile ionic bonds, they undergo protonation in aqueous acidic media to revert to their neutral phenolic forms. Evaluating this capacity for reprotonation serves as a robust validation of the ionic nature of the synthesized products. Since these phenolate salts contain a precise stoichiometric fraction of the parent polyphenol, isolating and quantifying the recovered phenolic fraction upon acidification offer definitive evidence of successful salt synthesis while validating the proposed molecular formulas and calculated molecular weights.

The recovery efficiencies for the inorganic naringin salts (Na, K, and Ca) were in close agreement with the theoretical values predicted by their stoichiometric formulas (Table 1). Complete quantitative conversion was marginally limited, which is attributed to competing tautomerization pathways and the structural influence of native glycosidic bonds. The structural identity and purity of the regenerated free naringin were confirmed via ^1^H NMR and FT-IR spectroscopic analyses (Figure S4 and S5)

**Table 1:** Naringin Quantification from the Salts by UV-Vis Spectrophotometer

Salt code	Naringin content theoretical [%]	Naringin quantified by UV/direct weighing [%]	Naringin Conversion [%]
Na-Naringin	89.79%	83.06%	92.50%
K-Naringin	83.55%	74.2%	88.81%
TBA-Naringin	54.65%	0	0
Ca-Naringin	88.79%	72.55%	81.71%

Conversely, the organic tetrabutylammonium (TBA) salt could not be successfully converted back to its parent acidic form. This distinct behavior is attributed to the pronounced steric hindrance imposed by the bulky, hydrophobic TBA cation, which effectively obstructs hydronium ion access to the phenolate target sites and prevents reprotonation. The step-by-step chemical mechanism detailing this acid-induced ring closure is depicted in Figure 5.

**Figure 5:**
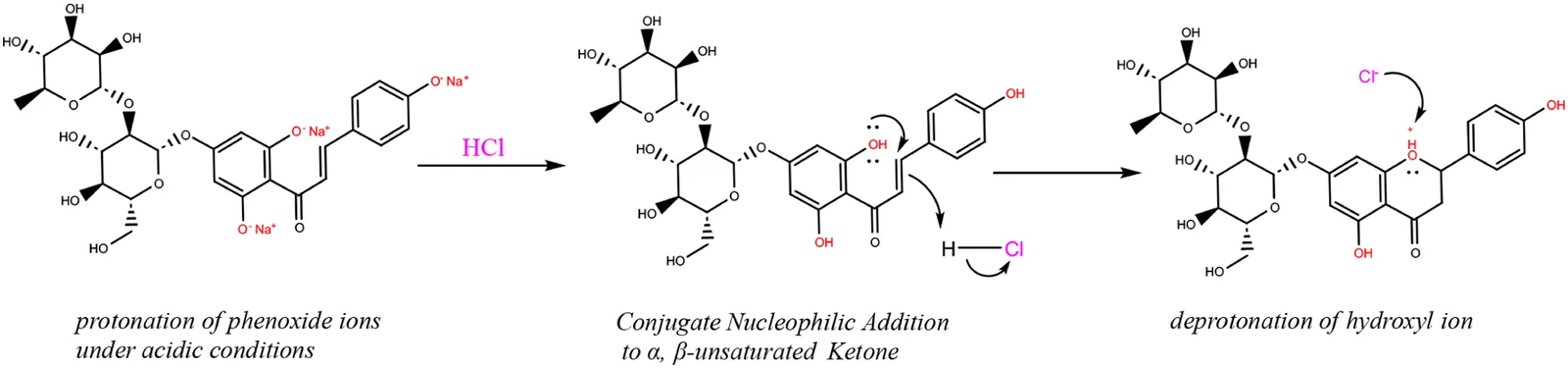
Proposed mechanism of the back generation reaction in naringin salts.

#### 3.2.4 Elemental Analysis

Elemental composition analysis (C, H, N, and O) was performed to elucidate the empirical formulas of the synthesized naringin salts and to quantify the number of coordinated water molecules within their respective crystal lattices. Minor deviations between the experimental and theoretical carbon and hydrogen weight fractions were observed across the metal phenolate series (Table 2). These variations are inherently characteristic of polyphenolic architectures like naringin, which feature multiple chemically distinct phenolic hydroxyl groups possessing unique p*K*_a_ values. Under basic conditions, competing deprotonation pathways can result in microheterogeneity regarding the exact coordination states or localized hydration levels across the bulk material. Consequently, the elemental data reflect a statistically dominant, average stoichiometric composition for the isolated salts. This closely aligns with the complex coordination equilibria frequently documented for flavonoid-metal complexes, confirming successful bulk modification.

**Table 2:** Elemental Analysis of Naringin and Its Salts Using CHN-O Elemental Analyses

N%		O%		H%		C%		
Exp.	Calc.	Exp.	Calc.	Exp.	Calc.	Exp.	Calc.	Proposed molecular formula
-	-	38.65	38.56	5.84	5.56	51.83	55.80	C_27_ O_14_H_32_
-	-	40.90	41.88	5.33	5.37	43.11	43.48	C_27_H_29_O_14_Na_3_ * 5.5H_2_O
-	-	36.52	37.58	5.08	4.83	42.45	42.23	C_27_H_29_O_14_K_3_ * 4H_2_O
-	-	36.84	36.22	4.82	4.78	43.11	43.08	C_27_H_31_O_14_CaOHCa_2_*2H_2_O
2.22	2.08	21.80	28.43	9.01	9.08	59.84	60.41	C_27_H_30_O_14_(C_16_H_36_N)_1.5_4H_2_O

#### 3.2.5 Thermal Analysis

The melting points of the naringin Na and K salts were >350 °C, which is significantly higher than the melting temperature of unmodified naringin (164–173 °C) and naringin-TBA salt (105–110 °C) (Table S1, Figure S6). Thermogravimetric analysis (TGA) of the naringin Na and K salts indicates a 12.5% weight loss between 70 and 150 °C, which can be attributed to the evaporation of lattice-bound water molecules.

### 3.3 Solubility Studies

The solubility profiles of the various naringin salts in Double Distilled Water (DDW), phosphate-buffered saline (PBS, pH 7.2), acidic buffer (pH 4), and Dulbecco’s modified Eagle medium (DMEM) media (containing 10% FBS and 1% penicillin/streptomycin (P/S)) are summarized in Figure 6 and Table S2. Naringin salts bearing alkali metal counterions (Na^+^/K^+^) exhibite good solubility in DDW, whereas Ca^2+^ salts show poor aqueous solubility. This reduced solubility of calcium salts can be attributed to the formation of oligomeric complexes in water and physiological media. In contrast, TBA naringin salts display increased lipophilicity due to the presence of bulky organic cations with long hydrocarbon chains, resulting in decreased solubility in PBS and DDW. Overall, naringin salts demonstrate moderate solubility in PBS and DMEM culture media. Notably, all alkali metal salts show substantially improved aqueous solubility compared to unmodified naringin.

**Figure 6:**
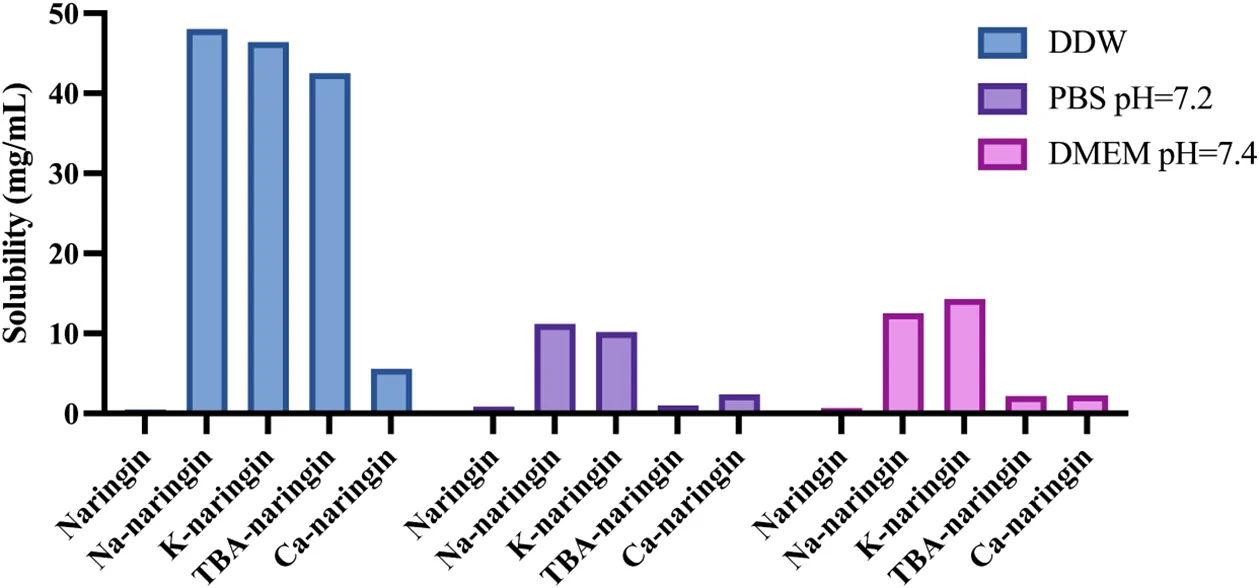
Solubility assessment of naringin salts in DDW, PBS, and DMEM.

### 3.4 MTT Assay

HepG2 cells were used as a preliminary and reproducible mammalian cell model to screen the cellular metabolic response to the newly synthesized naringin salts and identify potential cytotoxic effects. The MTT assay reveals time-dependent changes in metabolic activity following treatment with free naringin and its corresponding salts (Figure 7). Free naringin reduces metabolic activity relative to the untreated control, whereas several of its corresponding salts produce higher MTT signals, particularly after 48 h of exposure (Figure 7A). Qualitative microscopic examination also indicates an apparent increase in cell density in some salt-treated groups. Collectively, these findings suggest that selected naringin salts enhance the cellular metabolic activity and may support a growth-promoting cellular response. This is consistent with previous findings, demonstrating the growth-promoting potential of selected polyphenol salts in primary cell cultures.^14^ These observations provided a rationale for further evaluation of the synthesized salts in a more application-relevant cellular model.

**Figure 7:**
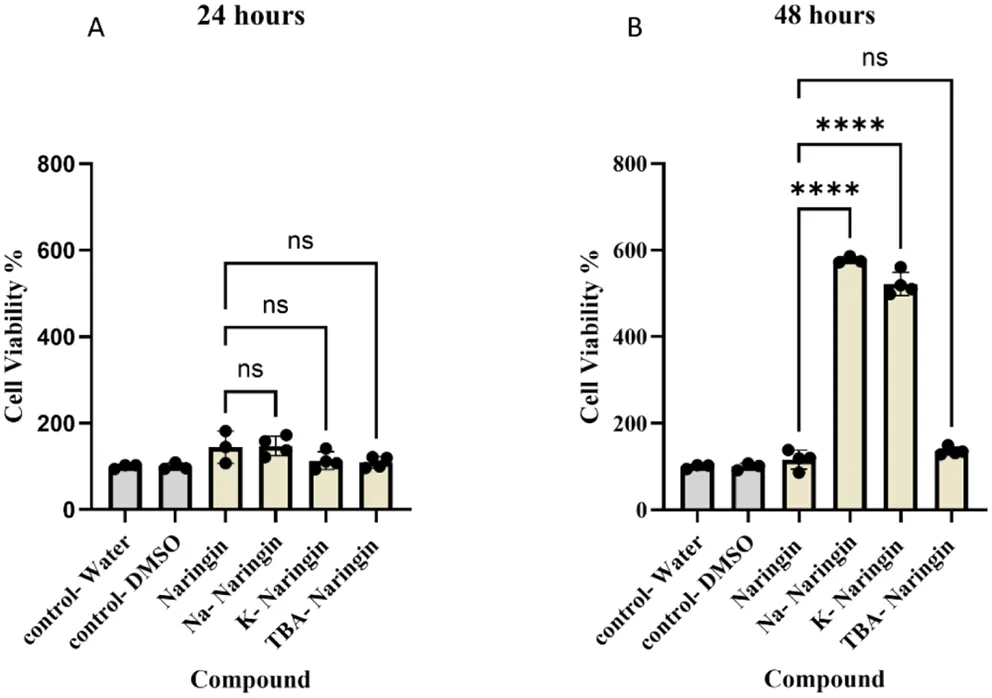
MTT-based metabolic activity of HepG2 cells treated with naringin and its phenolate salts. HepG2 cells (1 × 10^4^ cells/well) were treated with free naringin, Na-naringin, K-naringin, or TBA-naringin, each at a final concentration of 200 μM, for (A) 24 h and (B) 48 h. Untreated cells served as a control. Metabolic activity was determined using the MTT assay and expressed relative to the untreated control. Data are presented as mean ± SD from four independent biological replicates (*n* = 4). Statistical significance was determined using a two-tailed unpaired Student’s *t*-test relative to the untreated control: The p values are ****P < 0.0001, *P < 0.05.

### 3.5 Effect of Naringin Salts on Viability and Proliferation

After identifying the Naringin salts that showed increased metabolic activity, as determined by the MTT assay, we next evaluated their potential to support cell proliferation relative to commercially available FGF2 in the cultivated meat application. In a representative experiment (n = 2), we evaluated the viability of primary EChSCs under conditions using either standard FGF2 or Na/K-Naringin. The EChSCs cultured with Na-Naringin in the absence of FGF2 were almost 100% viable in cell culture, and cells treated with K-Naringin showed 100.15% viability relative to control (Figure 8A).

As a proof-of-concept, the cumulative population doubling levels (PDLs) of cells treated with Na-naringin or commercial FGF2 were monitored over 6 days. In this representative experiment (n = 2), Na-Naringin maintained cell viability and supported proliferative capacity, as reflected by the PDL values, at levels comparable to those observed with commercially available FGF2 (Figure 8B).

**Figure 8:**
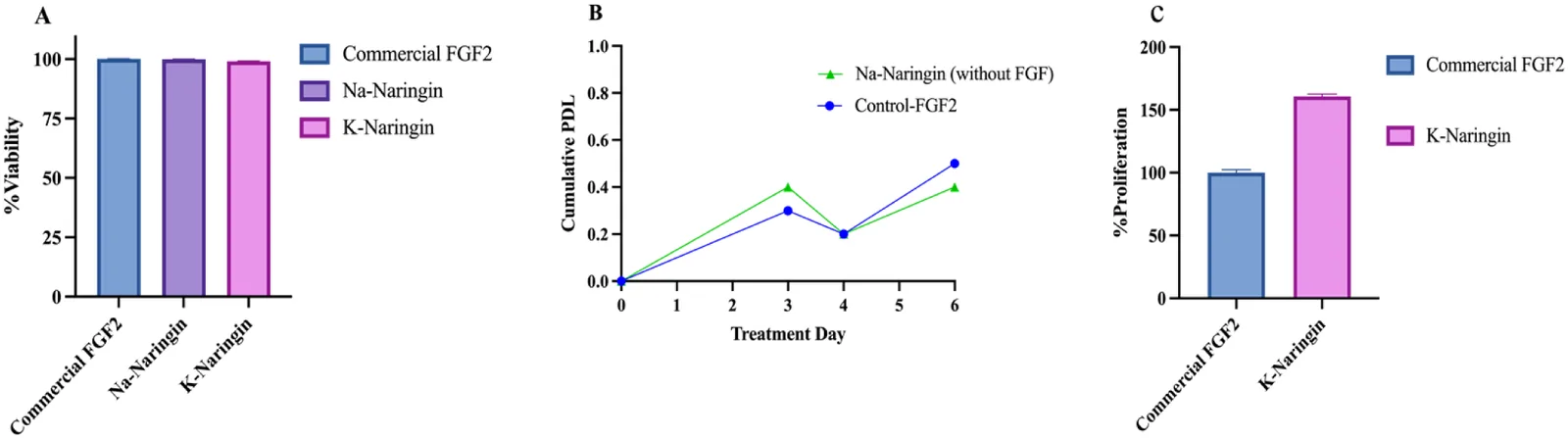
A) Comparison of percentage cell viability following treatment with commercial FGF2 or Na-Naringin/K-Naringin. Cells were treated for 3 days with either commercial FGF2 at 0.29 nM (blue, control), Na-Naringin (purple), or K-Naringin (pink) at 0.5 nM (*n* = 2). Viability was assessed and normalized to the FGF2-treated control, which was set to 100%. B) Accumulative population doubling levels (PDL) over time in cells treated with commercial FGF2 or Na-Naringin. Cells were cultured for 6 days in the presence of either commercial FGF2 (blue, control) or Na-Naringin (green). PDL, representing the number of population doublings cultured for 6 days (blue and green), was measured on days 3, 4, and populations cultured for 6 days (blue and green). C) Comparison of relative cell proliferation following treatment with commercial FGF2 or K-Naringin. Proliferation was assessed after 3 days and normalized to the FGF2-treated control (blue, 100 ). K-Naringin-treated cells (pink) showed a marked increase to 160.

Similar to Na-naringin, K-naringin maintained high cell viability in the absence of commercial FGF2. In addition, relative cell proliferation was assessed following treatment with either commercial FGF2 or K-naringin. In this preliminary experiment, a higher relative proliferation value was observed in K-naringin-treated cells compared with the commercial FGF2-treated reference condition (Figure 8C).

In a representative cell count experiment (n = 1), Na-Naringin-treated cells showed higher cell numbers than the commercial FGF2-treated reference condition at most time points until near the end of the experimental period. In contrast, K-Naringin supported initial increases in cell number but exhibited a reduction in growth rate after approximately one and a half weeks, resulting in lower cell numbers at later time points (Figure 9A). These preliminary observations suggest different growth profiles for Na-Naringin and K-Naringin over the experimental period. Na-Naringin-treated cultures showed relatively sustained cell numbers, whereas K-Naringin-treated cultures showed a reduction in cell numbers at later time points. In K-Naringin-treated cells, proliferation was initially observed. After about one and a half weeks, however, the rate of cell division decreased, leading to a reduced number of cells in the end of the experiment. In accordance with these findings, cells treated with Na-Naringin had considerably greater PDL values across all passages, indicating that Na-Naringin-treated cells showed higher PDL values across the monitored passages, whereas K-Naringin-treated cells showed lower PDL values at later stages of culture (Figure 9B).

**Figure 9:**
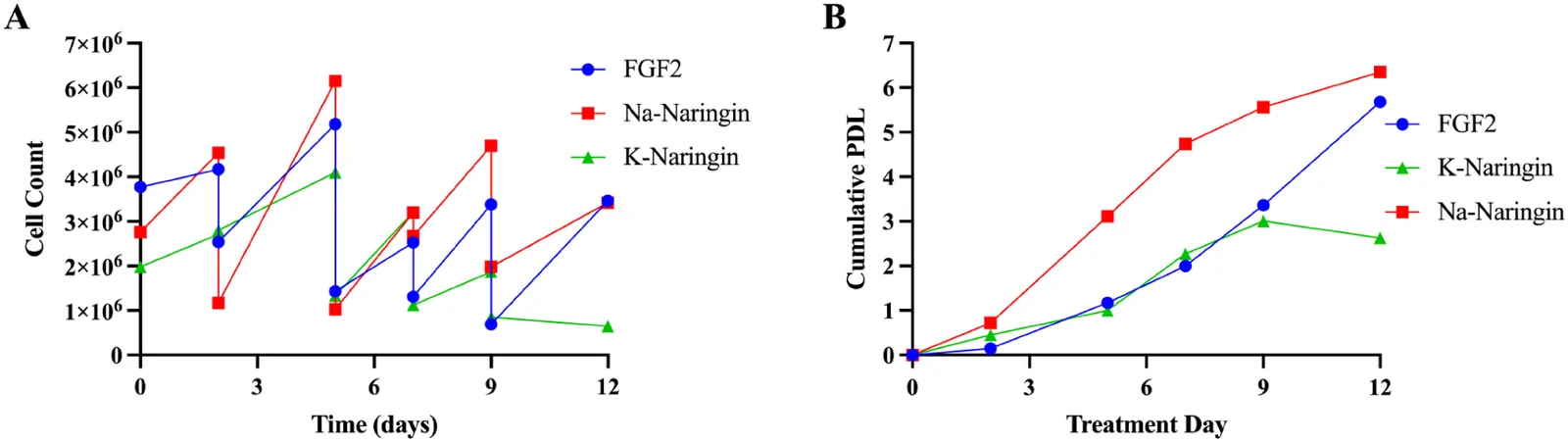
A) Cell growth under commercial FGF2, Na-Naringin, and K-Naringin treatment over 12 days. Cell growth was monitored over a 12 days period by quantifying cell numbers under treatment with commercial FGF2 (blue), Na-Naringin (red), or K-Naringin (green). Cell counts were obtained at each passage and used to evaluate changes in cell number over time (*n* = 1). B) Accumulative population doubling level (PDL) under commercial FGF2, Na-Naringin, and K-Naringin treatment over 12 days. Accumulative PDL was monitored over 12 days to assess long-term proliferative capacity under treatment with commercial FGF2 (blue), Na-Naringin (red), or K-Naringin (green) (*n* = 1). Population doublings were calculated from cell counts obtained at each passage.

Studies of cell growth kinetics suggested that Na-naringin may have the potential to support cell proliferation with values falling within the range observed for the commercial FGF2-treated reference condition.

Additionally, Na-naringin-treated cells showed higher values than the FGF2-treated reference condition across the tested conditions, suggesting that this phenolate salt may support the expansion phase (Figure S7). In addition, higher accumulated PDL values were observed in Na-naringin-treated cultures than in the FGF2-treated reference condition.

Taken together, these preliminary observations suggest that Na-naringin may have the potential to support growth performance and replicative longevity at levels comparable to those observed with FGF2 (Figure S8).

Cell proliferation kinetics (n = 1) upon treatment with both the commercial FGF2 preparation and the polyphenol Na-naringin were analyzed by flow cytometry of cells in control and treatment conditions at the time of seeding and at each time point before and after treatment.

In this single representative experiment (n = 1), Na-naringin-treated cells showed a proliferation profile that appeared similar to that observed under the commercial FGF2-treated reference conditions (Figure 10).

**Figure 10:**
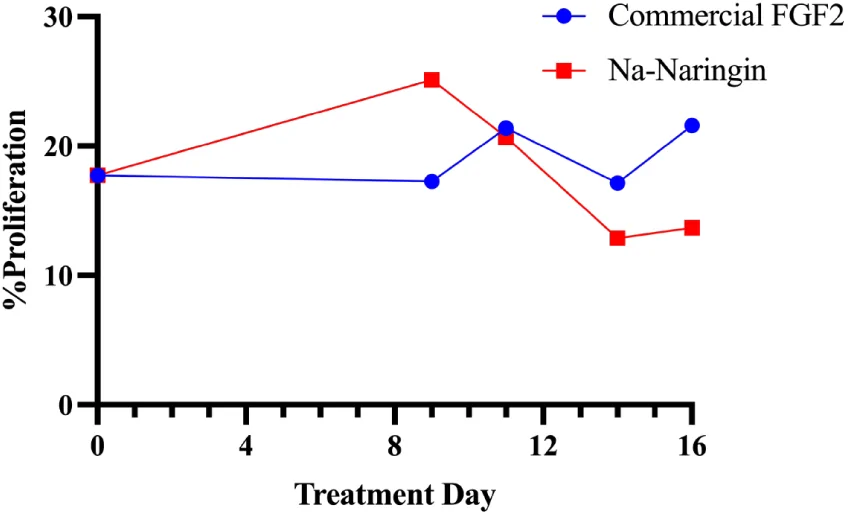
Comparison of cell proliferation under commercial FGF2 versus Na-Naringin treatment (*n* = 1). Cell proliferation was assessed by quantifying the proliferating fraction (S–M%) of the cell cycle using flow cytometry following Hoechst staining. Measurements were taken at multiple time points throughout the experiment up to day 16, starting on the day of seeding (Day 0).

On the basis of this proof of concept, *in vitro* experiments using EChSCs suggest preliminary observations that alkali naringin salts, particularly the sodium and potassium salts, may have the potential to support cell proliferation. These findings warrant further investigation of their possible applications in cultivated meat production. Future studies will focus on elucidating the molecular mechanisms underlying these observations and determining whether these salts could reduce the requirement for FGF2 or other growth factors, potentially contributing to the development of more cost-effective culture media.

## Supplementary Material



## Notes

Thermal analysis data, water-content estimation from elemental analysis and TGA, melting point measurements, a naringin calibration curve, additional NMR data, and supplementary cell growth and accumulated population doubling level (PDL) data comparing commercial FGF2 and Na-Naringin treatments (PDF)

## Abbreviations

^1^H NMR: Proton nuclear magnetic resonance
7-AAD: 7-Aminoactinomycin D
DDW: Double-distilled water
DMEM: Dulbecco’s modified Eagle medium
DMSO: Dimethyl sulfoxide
EChSCs: Embryonic chicken stem cells
FBS: Fetal bovine serum
FGF2: Fibroblast growth factor 2
FGFRs: Fibroblast growth factor receptors
FT-IR: Fourier-transform infrared spectroscopy
GPFs: Growth-promoting factors
GRAS: Generally recognized as safe
HepG2: Human hepatocellular carcinoma cells
HSQC: Heteronuclear single quantum coherence
K-Ng: Potassium naringenin
MTT: 3-(4,5-Dimethylthiazol-2-yl)-2,5-diphenyltetrazolium bromide
Na-Q: Sodium quercetin
P/S: Penicillin/streptomycin
PBS: Phosphate-buffered saline
PDL: Population doubling level
SM: SuperMeat
TBA: Tetrabutylammonium
TBAOH: Tetrabutylammonium hydroxide
TLC: Thin-layer chromatography
