# Supplementary material for: Synthesis and Characterization of Naringin Phenolate Salts for Cultivated Meat
Source: J Agric Food Chem. 2026 Sep 24;74(39):31127–36. doi: 10.1021/acs.jafc.6c09167 (PMC13651613; doi:10.1021/acs.jafc.6c09167)
Supplement: Supplementary file 1 [file jf6c09167_si_001.pdf]

Synthesis and Characterization of Naringin Phenolate Salts for Cultivated Meat

Matilda Dirawy[1‡], Muhammad Abdel-Haq[1‡], Sarah E. Hasenson[2], Sara Mahajna[1], Mohamed Mahameed[1], M. N. V. Ravi Kumar[3], Meenakshi Arora[3], Abraham J. Domb[1*]

1.Institute for Drug Research, School of Pharmacy, Faculty of Medicine, The Hebrew University of Jerusalem, Jerusalem 91120, Israel

2. SuperMeat Co., The Essence of Meat Ltd, Pinhas Sapir 3, Nes Ziona 7403626, Israel

3. Department of Translational Science and Medicine, College of Community Health Sciences and The Center for Convergent Bioscience and Medicine (CCBM), The University of Alabama, Tuscaloosa 35401, USA

‡ Equal Contribution

*Correspondence author email: avid@ekmd.huji.ac.il

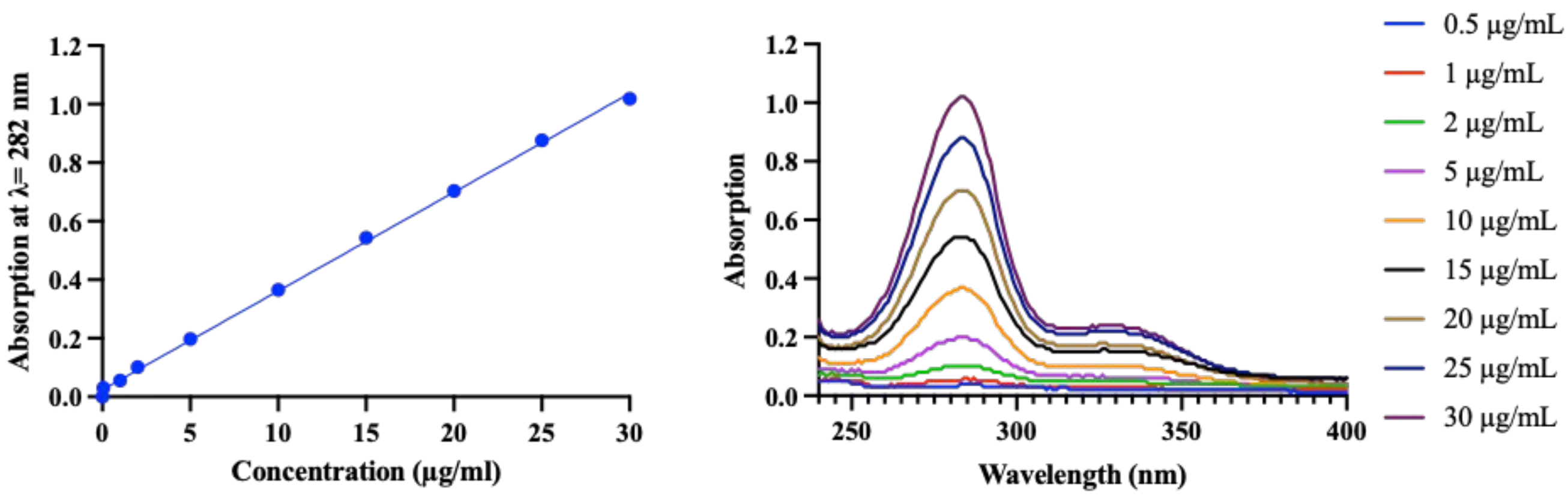

Figure S1: Naringin calibration curve in ethanol .

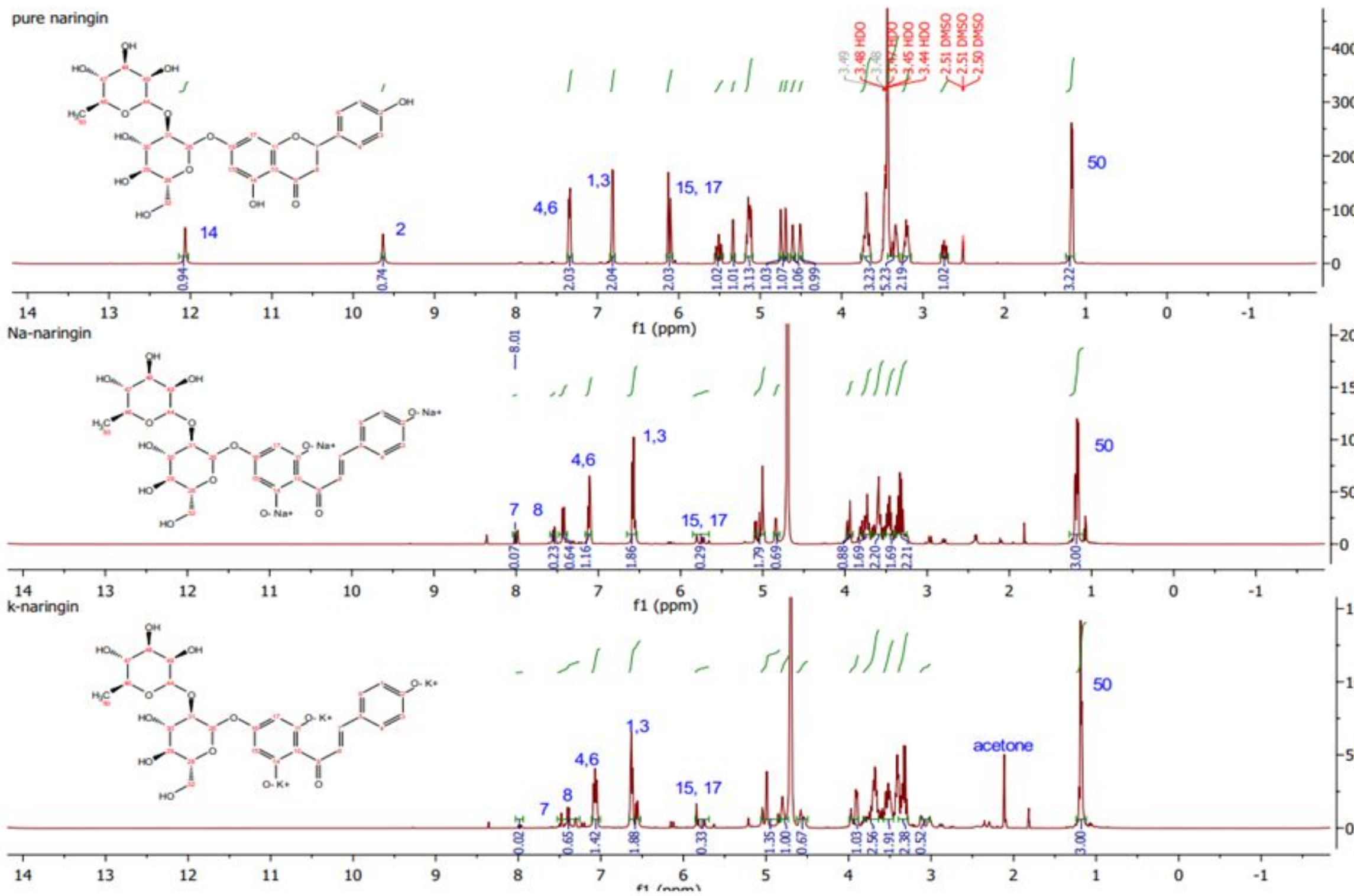

Figure S2. ¹HNMR spectra of naringin (dmso-d6) and Na/k naringin in D2O

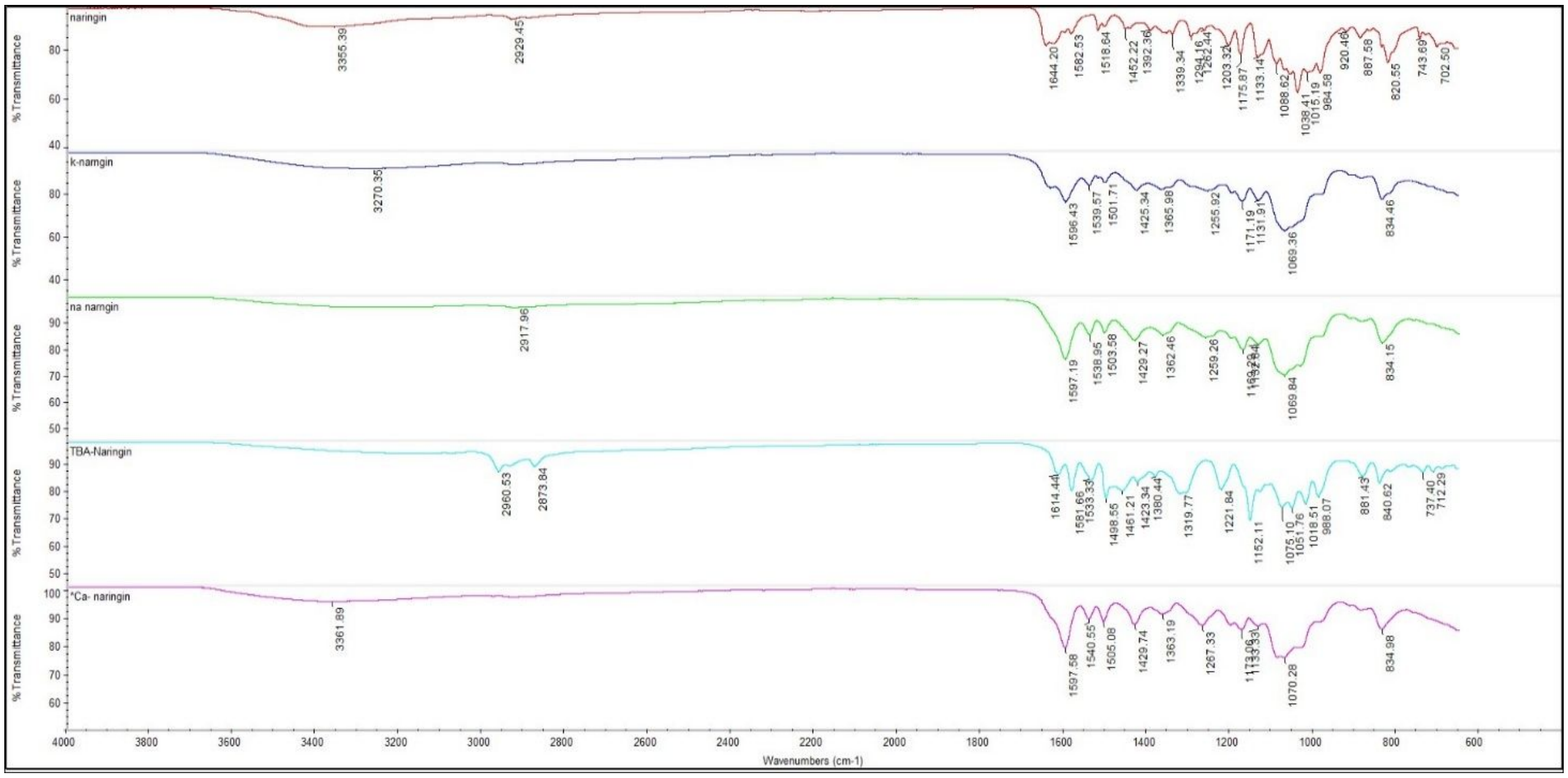

Figure S3. FT-IR spectra of naringin and Na/k/Ca/TBA salts

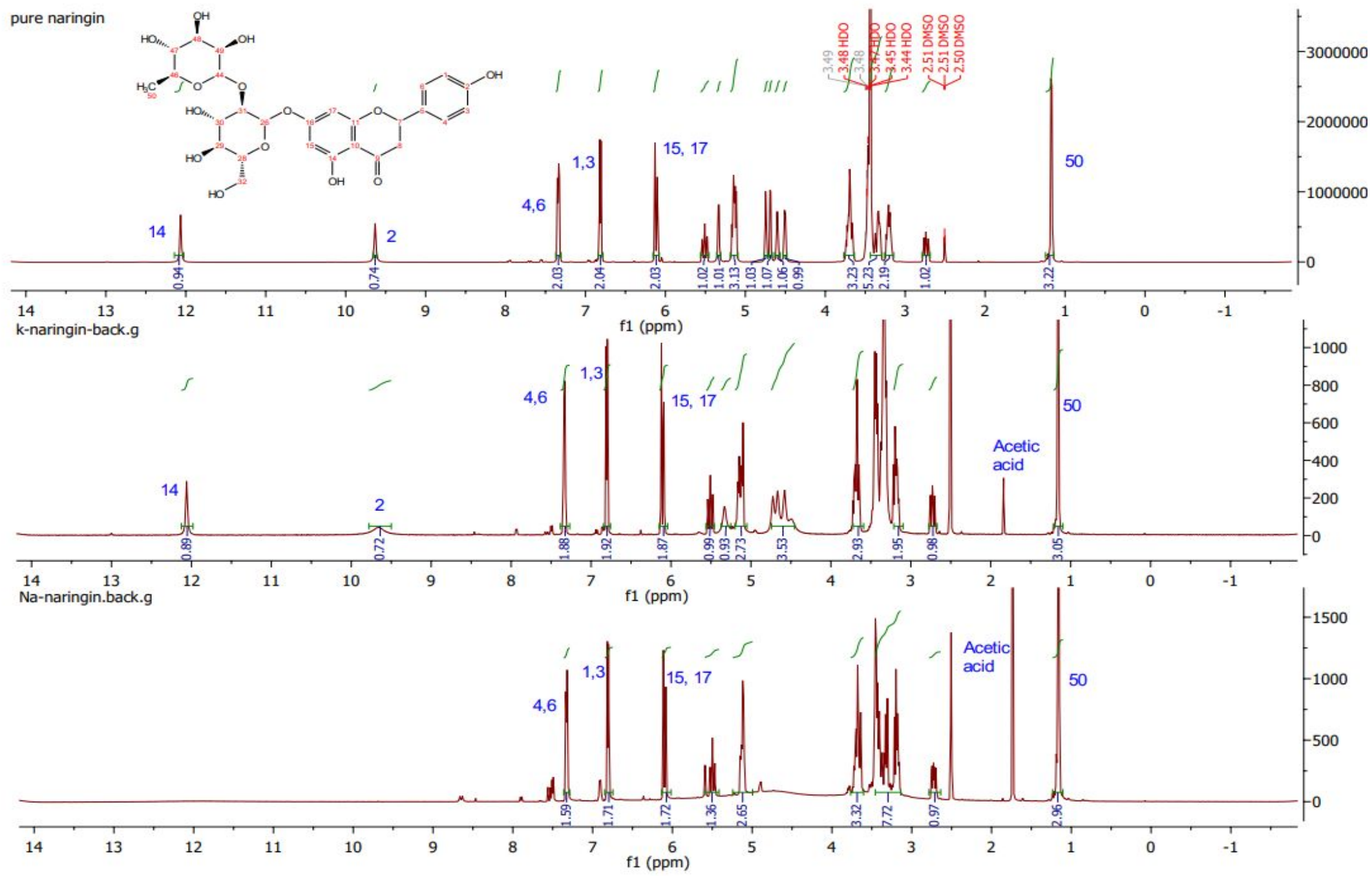

Figure S4. HNMR spectra of naringin and the recovered products from its salts

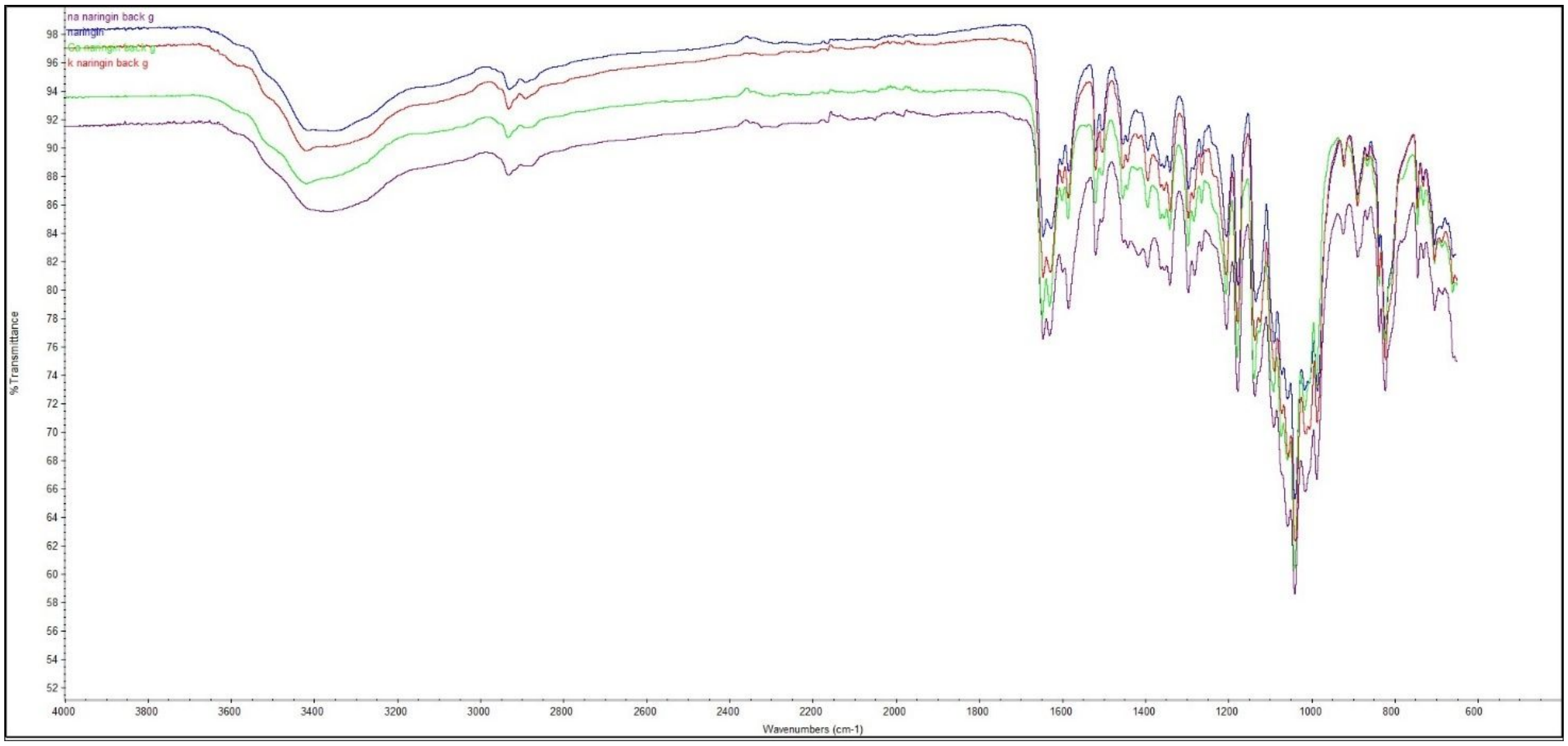

Figure S5: FTIR spectra of naringin and the recovered products from its salts.

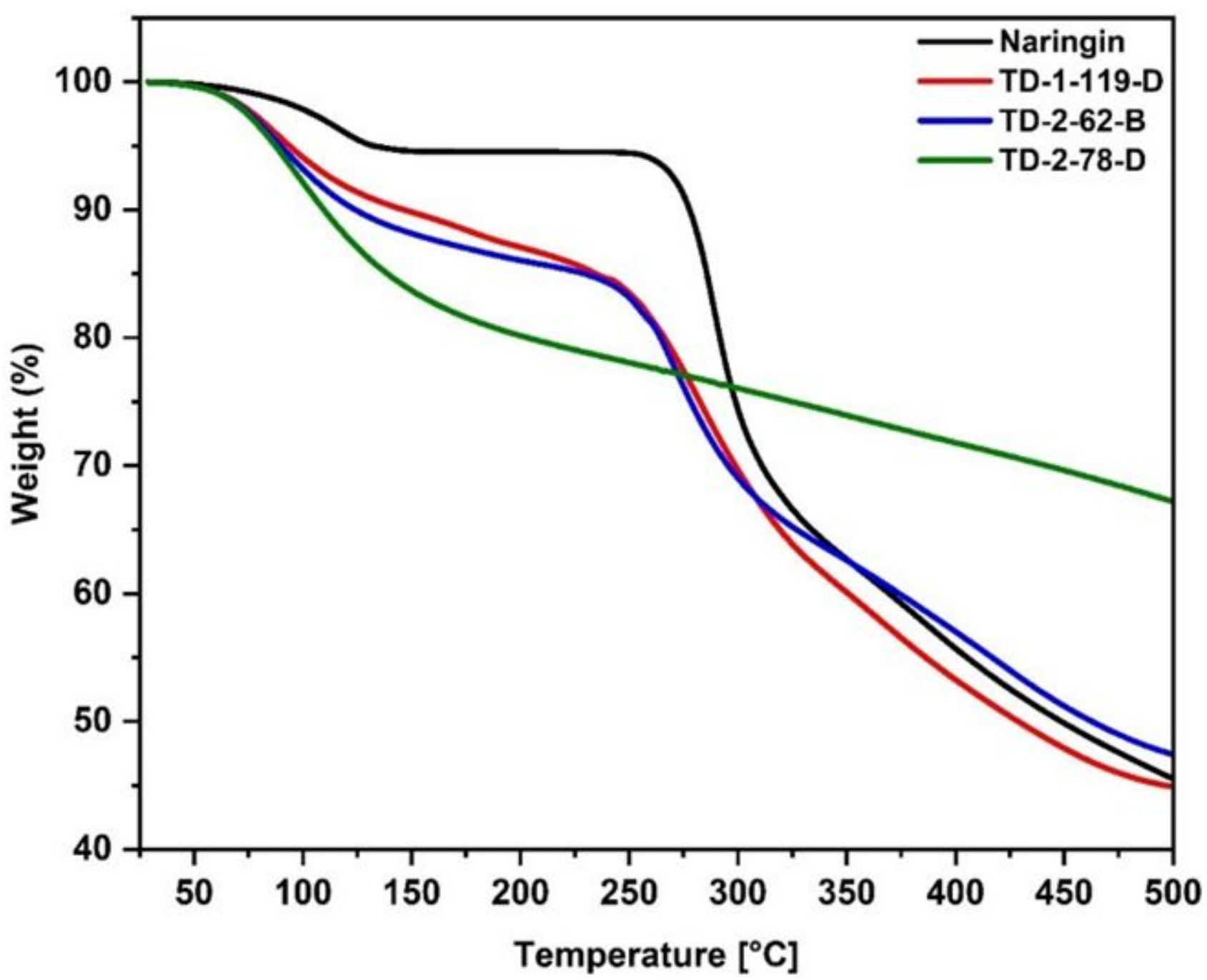

Figure S6: Thermogravimetric Analysis of various Naringin salts

Table S1. Thermal analysis Data of various Naringin salts

Sample	H_2O percent from elemental analysis	H_2O percent from TGA	Onset/offset temp. (TGA)	Melting point Exp.
Naringin	0%	5.5%	Onset=274.6 ^oC Offset=307.16 ^oC	164-173 ^oC
Na-Naringin	13.2%	12.5%	Onset=255 ^oC Offset=293.35 ^oC	>360 ^oC (m.p instrument)
K-Naringin	9.40%	12.85%	Onset=256.29 ^oC Offset=334 ^oC	>360 ^oC (m.p instrument)
TBA-Naringin	1.66%	NA*	Onset=70.84^oC Offset=136.75 ^oC	105-110 ^oC (m.p instrument)

*NA- not applicable

Table S2 . Solubility of naringin salts and its salts in DDW, PBS and DMEM

**Salt code**	**Solubility in DDW**	**Solubility in PBS pH=7.2**	**Solubility in DMEM pH=7.4**
**naringin**	0.5mg/ml	0.85mg/ml	0.7mg/ml
**Na-Naringin**	48mg/ml	11.2mg/ml	12.5mg/ml
**K-Naringin**	46.4mg/ml	10.2mg/ml	14.3mg/ml
**TBA-Naringin**	42.5mg/ml	1mg/ml	2.2mg/ml
**Ca-Naringin**	5.6mg/ml	2.4mg/ml	5.28mg/ml

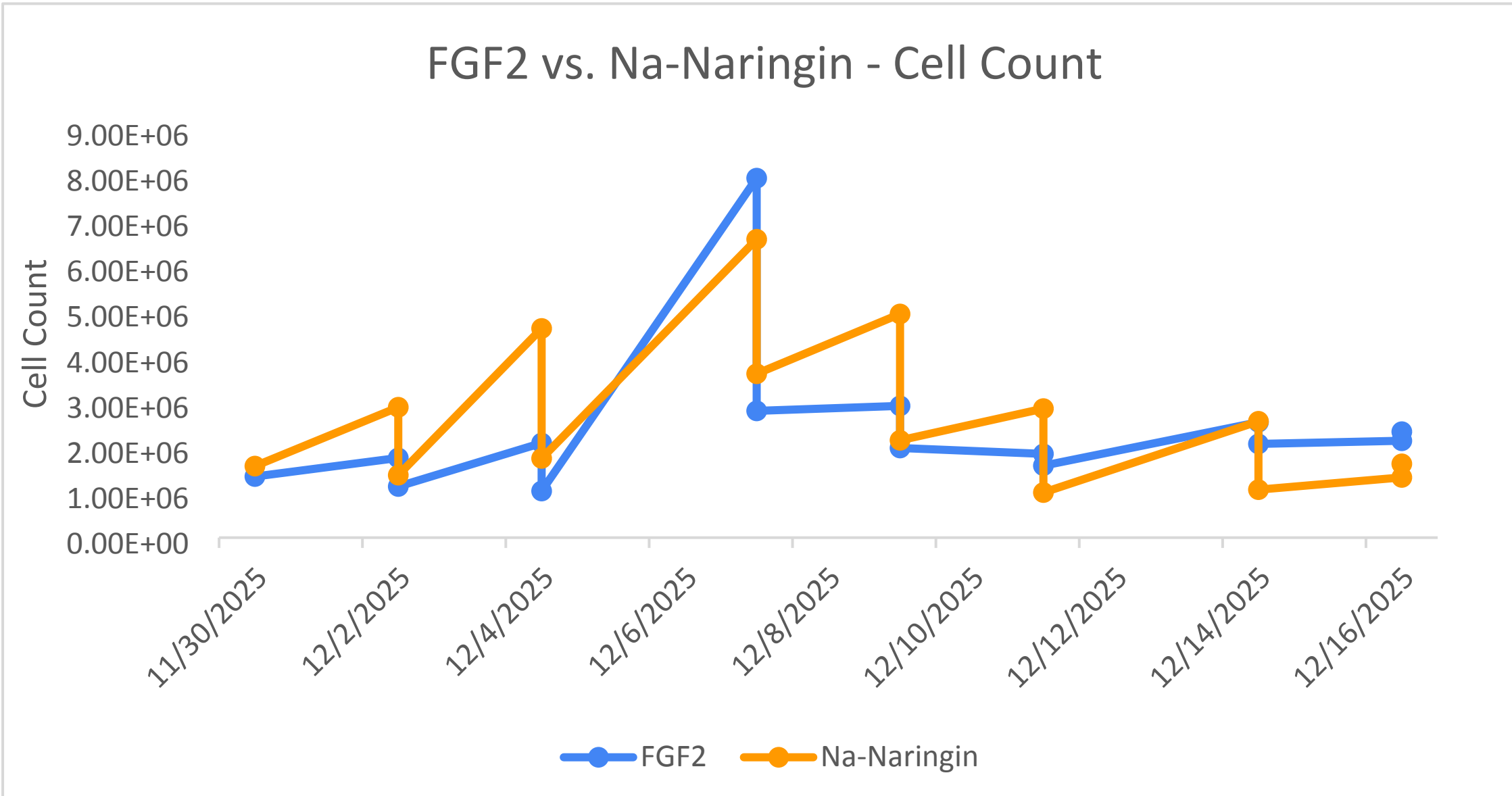

Figure S7.Effect of commercial FGF2 versus Na-Naringin on cell growth.

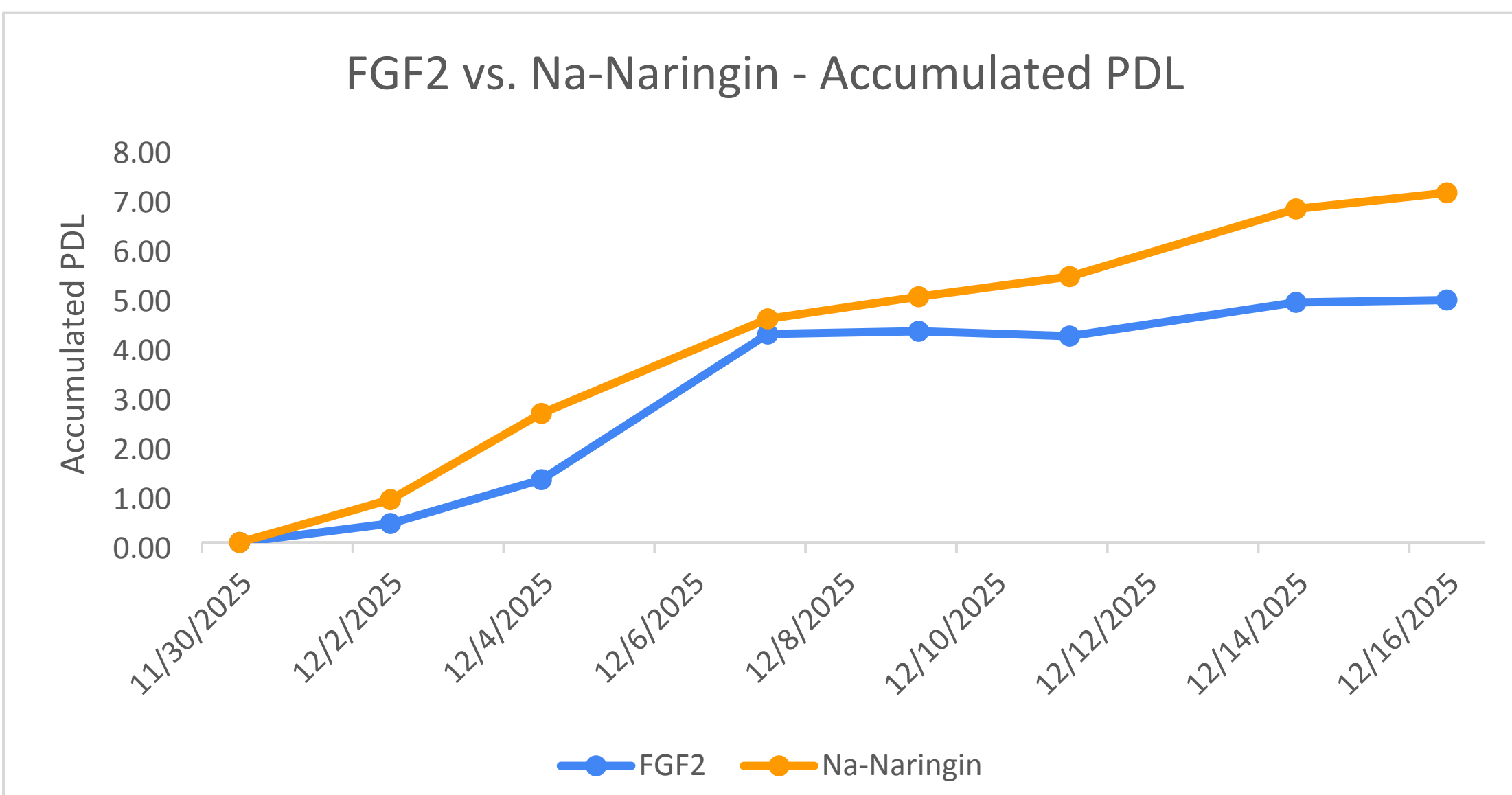

Figure S8. Accumulative population doubling level (PDL) under commercial FGF2 or Na-Naringin treatment.
